# In‐Hospital Cardiac Arrest Detection Performance Analysis and Comparison on Effective Feature Selection

**DOI:** 10.1002/clc.70395

**Published:** 2026-06-30

**Authors:** Tianxin Jiang, Junbiao Liu, Dinghan Hu, Mengyuan Diao, Jiuwen Cao

**Affiliations:** ^1^ Machine Learning and I‐Health International Cooperation Base of Zhejiang Province and School of Automation Hangzhou Dianzi University Hangzhou Zhejiang China; ^2^ Department of Critical Care Medicine, Affiliated Hangzhou First People's Hospital Zhejiang University School of Medicine Hangzhou Zhejiang China

**Keywords:** correlation analysis, feature selection, in‐hospital cardiac arrest, machine learning

## Abstract

**Background:**

How to reduce the occurrence of in‐hospital cardiac arrest (IHCA), screen potential IHCA patients, and advance the treatment of IHCA are urgent problems to be solved in clinic. In this study, we tried to develop a model to predict whether patients will develop IHCA based on the data of patients who have just been admitted to hospital and evaluate the influence of different feature selection methods on machine learning (ML) models.

**Methods and Results:**

A total of 25 149 patients were included in the study; 320 developed IHCA. We chose three feature selection methods (Student's *t*‐test and Chi‐square test, regression analysis and correlation analysis) and four ML models (AdaBoost, XGBoost, Random Forest, and Logistic Regression). Each ML model was trained and evaluated using raw and feature‐selected data; as a result, we got 16 models. AUROC, AUPRC, accuracy, recall, precision, and specificity are used to evaluate the model. The XGBoost model has the best performance with an AUROC of 0.987 (95% CI 0.984–0.988), an AUPRC of 0.763, an accuracy of 0.992, a recall of 0.695, a precision of 0.723, and a specificity of 0.996. The most significant predictors are age, albumin, sinus arrhythmia, activated partial thromboplastin time, and protein.

**Conclusions:**

Different feature selection methods have different effects on different ML models. The predictive model developed using the XGBoost algorithm is the best predictor of whether patients will develop IHCA.

## Graphical abstract

We systematically compared three feature selection strategies and four machine learning algorithms for in‐hospital cardiac arrest prediction. XGBoost achieved the best performance, and key predictive factors were identified through feature selection and SHAP analysis for early clinical risk stratification.

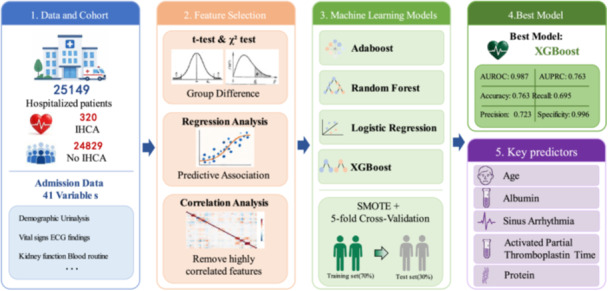

## 1 Introduction

In‐hospital cardiac arrest (IHCA) is defined as the sudden cessation of cardiac ejection in hospitalized patients, resulting in severe ischemia and hypoxia of vital organs, and ultimately leading to death. IHCA is a life‐threatening adverse event associated with an extremely high mortality rate [1, 2]. Given the persistently low survival rate of IHCA, it imposes a major public health burden and entails substantial consumption of medical resources, rendering the early prediction and prevention of IHCA clinically crucial. Clinical deterioration frequently precedes IHCA [3], and a substantial proportion of IHCA events are considered preventable or avoidable in retrospective reviews [4, 5]. How to reduce the incidence of IHCA, identify patients at high risk, and optimize timely therapeutic interventions represents an urgent clinical challenge.

Over the past decade, many countries have built IHCA's early‐warning scoring system. At present, the commonly used scoring systems are National Early Warning Score (NEWS) [6, 7], Modified Early Warning Score (MEWS) [7, 8], and electronic Cardiac Arrest Risk Triage (eCART) [9]. However, growing evidence has revealed critical flaws in these conventional scoring systems. NEWS predicts cardiac arrest in patients with acute coronary syndrome with an AUC of 0.74, specificity of 0.81, and sensitivity of 0.55. Creutzburg et al. reported that implementation of NEWS failed to reduce the incidence of IHCA in general wards [6, 7]. MEWS predicts cardiac arrest in patients with acute coronary syndrome with an AUC of 0.73, a specificity of 0.93, and a sensitivity of 0.44. But Kim et al. further demonstrated that MEWS alone is insufficient for reliable IHCA prediction [7, 10]. The eCART system shows a higher AUC of 0.801 [11], but its performance is limited by long intervals between biochemical tests and reliance on historical values rather than real‐time admission data. Collectively, traditional warning scores rely on simple linear combinations of a small set of parameters and cannot capture complex non‐linear relationships in multi‐modal clinical data [12].

With the continuous advancement of Artificial Intelligence (AI), machine learning (ML) frameworks have been increasingly applied in hospital emergency settings, and accumulating evidence demonstrates that such technologies can assist in early warning, prognosis assessment, and clinical decision‐making [13, 14, 15]. Recent studies confirm that ML models outperform conventional scoring systems for IHCA prediction, with AUROCs above 0.85 [16, 17, 18]. Nevertheless, several important limitations remain in current research. While feature selection has been adopted in some IHCA prediction models, most existing studies rely on only one feature selection strategy and lack head‐to‐head comparisons across methods. Many existing studies lack standardized feature selection procedures and often do not provide clear rationales for the variables included in model construction. Physiological parameters are frequently incorporated directly into models without prior screening, which may lead to redundant features, multicollinearity, and reduced model stability and interpretability. Attin et al. [12] highlighted that no existing scoping review has comprehensively evaluated feature selection in ML‐driven IHCA prediction, and the lack of standardized feature selection and reporting represents a critical obstacle to further development.

Furthermore, the significant individual heterogeneity of IHCA patients further complicates early prediction and remains inadequately addressed in most existing studies [19]. Although feature selection is a standard component of ML pipelines, its application in IHCA prediction remains inconsistent and has rarely been compared across different methods. Therefore, identifying consistent predictive factors through systematic feature selection is critical for developing a clinically practical early warning model for IHCA. The purpose of this study is to analyze and screen the collected features, evaluate the influence of various feature selection methods on the final model, and identify the key predictors of IHCA, with the aim of achieving a more parsimonious and effective prediction model.

## 2 Methods

### 2.1 Data Source

The data used in this study are from the database of Hangzhou First People's Hospital. This database records the demographic characteristics of patients and the data of various examinations they participated in. The data that support the findings of this study are available from the corresponding author upon reasonable request. The data are not publicly available due to privacy.

### 2.2 Patient Population

The study used patient data from 2017 to 2020 and collected data from when they were first admitted. Inclusion criteria: (1) age > 18 years old; (2) patients with return of spontaneous circulation (ROSC) after CPR for IHCA. Exclusion criteria: (1) Patients who are unconscious for other reasons; (2) malignant tumors and other chronic diseases end in stage; (3) patients with incomplete data collection from cardiac arrest to ICU admission more than 24 h ago.

Three hundred and eighty‐two patients with IHCA were screened. As the database has missing values, patients with incomplete routine blood or coagulation data were excluded from the database for convenience and higher accuracy of subsequent data filling. Finally, 320 patients with IHCA were included in the study.

At the same time, 24 829 patients without cardiac arrest (all with routine blood and coagulation function data) were randomly selected from the entire hospital database for comparison.

### 2.3 Variables Examined and Data Processing

Members of the cardiac advisory committee determine variables included in this study. Initially, 56 candidate variables were collected, including sex, age, complete blood count indicators (*n* = 5), coagulation function indicators (*n* = 6), and 43 additional variables with missing data. Since missing data were widely present with varying rates across variables, we first evaluated how to handle missing values, including variable deletion or imputation, and determined an appropriate cutoff for the missing rate.

To select a robust imputation strategy, we conducted a comparative experiment on multiple missing value imputation algorithms. A complete subset without any missing values (3214 patients, 35 features) was extracted from the original data set. We then artificially generated three typical missing mechanisms: missing completely at random (MCAR), missing at random (MAR), and missing not at random (MNAR). To simulate realistic clinical missing scenarios, a mixed missing pattern (MCAR: MAR: MNAR = 4:4:2) was applied at four missing levels (20%, 40%, 60%, and 80%). Six imputation methods were evaluated: mean imputation, linear interpolation, multiple imputation by chained equations (MICE), k‐nearest neighbor (KNN), matrix completion imputation (MCI), and Bayesian imputation. Model performance was assessed using the AUC of random forest (RF) classifiers. The model built on the original complete non‐missing data set achieved a benchmark AUC of 0.830. As shown in Table 1, values in parentheses represent the absolute difference between the AUC of each imputation method and the 0.830 benchmark. KNN imputation yielded AUC values closest to those of the complete data and maintained high stability across all missing rates. Therefore, KNN was confirmed as the optimal imputation method for this study.

**Table 1:** Model performance (AUC) of different imputation methods under mixed missing patterns.

Missing rate	Mean	Linear	MICE	KNN	MCI	Bayes
20%	0.802 (0.028)	0.731 (0.099)	0.851 (0.021)	0.785 (0.045)	0.806 (0.024)	0.851 (0.021)
40%	0.626 (0.204)	0.884 (0.054)	0.722 (0.108)	0.822 (0.008)	0.765 (0.065)	0.722 (0.108)
60%	0.759 (0.071)	0.912 (0.082)	0.827 (0.003)	0.801 (0.029)	0.707 (0.123)	0.827 (0.003)
80%	0.770 (0.060)	0.884 (0.054)	0.686 (0.144)	0.798 (0.032)	0.779 (0.051)	0.686 (0.144)

**Figure 1:**
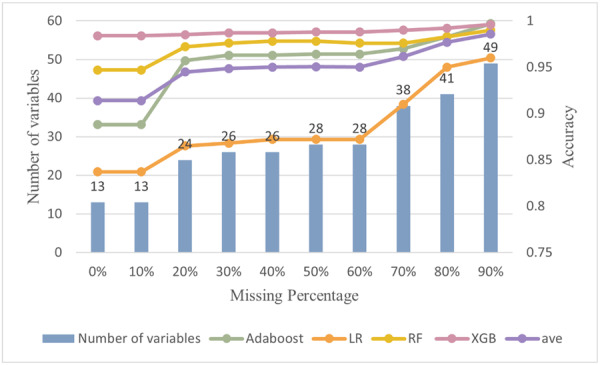
Relationship between variable missing percentage, number of retained variables, and average model accuracy.

With the optimal imputation method determined, we proceeded to perform KNN imputation on the original data set and further verify the appropriate missing rate cutoff. We first implemented the KNN imputation workflow as follows: since the patient data set we screened carried no missing values in gender, age, five complete blood count indicators, and six coagulation function indicators, we calculated Euclidean distances based on these 13 fully observed variables and used them to perform KNN imputation for the remaining features. To determine the optimal missing rate cutoff, we then filtered variables according to their missing rate in the raw data set and conducted preliminary model training to compare classification performance. As illustrated in Figure 1, the *x*‑axis represents the missing rate threshold for variable inclusion, the left *y*‑axis indicates the number of variables retained under the corresponding missing rate cutoff, and the right *y*‑axis shows the average classification accuracy of four mainstream ML models: (1) Adaptive Boosting (AdaBoost), (2) Extreme Gradient Boosting (XGBoost), (3) RF, and (4) Logistic Regression (LR). The blue bars in the figure correspond to the count of variables retained at each missing percentage cutoff. By comparing models trained on raw data without imputation and models trained after KNN imputation, we verified that missing value imputation was indispensable for stable model performance, and that using only the 13 fully observed variables yielded unsatisfactory classification accuracy. After a comprehensive evaluation, an 80% missing rate was adopted as the cutoff threshold in this analysis, as this threshold achieved the optimal balance between the number of included variables and overall model performance.

As illustrated in Figure 1, we performed KNN imputation as follows: since the patient data set we screened carried no missing values in gender, age, five complete blood count indicators, and six coagulation function indicators, we calculated Euclidean distances based on these 13 variables and used them to perform KNN imputation for the remaining features. We then filtered variables according to their missing rate in the raw data set and conducted preliminary model training to compare classification accuracy. By comparing models trained on raw data without imputation and models trained after KNN imputation, we verified that missing value imputation was indispensable, and that using only the 13 fully observed variables yielded unsatisfactory classification performance. After a comprehensive evaluation, an 80% missing rate was adopted as the cutoff threshold in this analysis.

Finally, a total of 41 variables (32 continuous, 9 categorical) were retained: sex, age, liver function indicators (*n* = 10), urinalysis indicators (*n* = 5), coagulation function indicators (*n* = 6), renal function indicators (*n* = 3), myocardial injury indicator (*n* = 1), vital signs (*n* = 4), complete blood count indicators (*n* = 5), and electrocardiogram (ECG) indicators (*n* = 5). The primary patient outcome was the incidence of IHCA, which is defined as an event of cardiac arrest occurring in the hospital.

## 3 Feature Selection

### 3.1 Student's *t*‐Test and Chi‐Square Test

Student's *t*‐test and Chi‐square test are two common and basic hypothesis testing methods, which can be used for medical data analysis. Student's *t*‐test uses t‐distribution theory to infer the probability of a difference, so as to compare whether the difference between two averages is significant. The chi‐square test determines whether two categorical variables are related by chi‐square statistics.

Continuous variables are presented as the mean and standard deviation and compared between groups by using Student's *t*‐test. Nominal variables are expressed in frequency and fraction and analyzed by using the chi‐square test. In general, the *p *< 0.05 indicates a significant difference between the two groups of data. A total of 32 features with *p* < 0.05 are screened (24 continuous and 8 categorical) (Table 2).

**Table 2:** Sample characteristics of the study population.

Variables	All patients	*p* value
	(*n* = 25 149)	
Age	58.13 ± 18.72	< 0.001
Male sex	11 670 (46.4%)	< 0.001
Liver function		
ALT	40.86 ± 64.68	0.465
GGT	80.54 ± 146.76	0.025
ALP	97.74 ± 110.73	0.004
TP	63.19 ± 6.63	< 0.001
ALB	34.97 ± 4.90	< 0.001
GLOB	28.23 ± 5.21	0.325
TBIL	19.20 ± 39.83	0.023
DBILI	10.01 ± 29.33	0.021
IBIL	9.18 ± 11.69	0.05
B‐AMY	68.26 ± 52.37	0.406
Urinalysis		
PH	6.15 ± 0.67	< 0.001
PRO	3273 (13.0%)	< 0.001
KET	1998 (7.9%)	< 0.001
RBCU	3796 (15.1%)	< 0.001
WBCU	6246 (24.8%)	0.015
Blood coagulation function		
PT	11.36 ± 2.08	< 0.001
INR	1.02 ± 0.20	< 0.001
APTT	27.86 ± 4.08	< 0.001
TT	16.48 ± 3.76	< 0.001
FG	3.15 ± 1.15	< 0.001
D‐D	1633.66 ± 4608.00	< 0.001
Kidney function		
CR	8.33 ± 5.32	< 0.001
Urea	5.23 ± 3.64	< 0.001
UA	258.01 ± 109.19	< 0.001
Indicators of myocardial injury		
BNP	2315.17 ± 5747.35	< 0.001
Vital signs		
NIBP	129.16 ± 20.13	0.432
RESP	19.39 ± 1.81	< 0.001
TEMP	36.84 ± 0.48	0.83
SPO2	97.84 ± 2.74	0.019
Blood routine examination		
WBC	7.23 ± 5.92	< 0.001
NEUT	68.39 ± 13.46	< 0.001
LYM	1.45 ± 2.93	0.024
HGB	122.85 ± 24.52	< 0.001
PLT	202.34 ± 79.98	< 0.001
Electrocardiogram		
Sinus arrhythmia	13 542 (53.8%)	< 0.001
Ectopic cardiac rhythm	1733 (6.9%)	< 0.001
Conduction block	1980 (7.9%)	< 0.001
Pre‐excitation syndrome	25 (0.1%)	0.297
Pacemaker heart rate	119 (0.5%)	< 0.001

Abbreviations: ALB, albumin; ALP, alkaline phosphatase; ALT, sample characteristics of the study population; APTT, activated partial thromboplastin time; B‐AMY, B‐amylase; BNP, brain natriuretic peptide; CR, creatinine; DBIL, total bilirubin; D‐D, D‐Dimer; FG, fibrinogen; GGT, gamma glutamyl transpeptidase; GLOB, globulin; HGB, hemoglobin; IBIL, indirect bilirubin; INR, international normalized ratio; KET, ketone bodies; LYM, lymphocyte; NEUT, neutrophil; NIBP, noninvasive blood pressure; PLT, platelet; PRO, protein; PT, prothrombin time; RBCU, red blood cell‐urine; SPO2, oxyhemoglobin saturation; TBIL, total bilirubin; TP, total protein; TT, thrombin time; UA, uric acid; WBC, white blood cell; WBCU, white blood cell‐urine.

### 3.2 Regression Analysis

Regression analysis is also a common method for medical analysis, which reflects the relationship between one dependent variable and several independent variables. In this paper, binary logistic regression is used. The advantage of logistic regression analysis is that the independent variables can be continuous or discrete in the statistical analysis process, which has significant advantages for processing multimodal medical data. Besides, SPSS is used for regression analysis of the data. Table 3 shows the results of the regression analysis. The *p* < 0.05 indicates that the independent variable has a significant impact on the dependent variable (i.e., whether the patient develops IHCA). The OR value is the ratio of the exposed to non‐exposed number in the case group and the exposed to non‐exposed number in the control group. A total of 15 features (12 continuous and 3 categorical) with *p* < 0.05 were screened.

**Table 3:** Results of regression analysis.

Variables	*B*	Standard error	Wald	*p* value	OR‐value	95% CI
Sex	−0.152	0.161	0.893	0.345	0.859	0.627–1.177
Age	0.034	0.006	32.179	0.000	1.035	1.023–1.047
PT	4.289	0.459	87.142	0.000	72.915	29.628–179.444
INR	−45.945	4.951	86.104	0.000	0.000	0.000–0.000
APTT	0.042	0.011	15.091	0.000	1.043	1.021–1.065
TT	−0.005	0.015	0.094	0.759	0.996	0.967–1.025
FG	−0.047	0.063	0.554	0.457	0.954	0.844–1.079
D‐D	0.000	0.000	5.364	0.021	1.000	1.000–1.000
WBC	0.013	0.008	2.860	0.091	1.013	0.998–1.029
NEUT	−0.010	0.006	2.658	0.103	0.990	0.978–1.002
LYM	−0.038	0.063	0.357	0.550	0.963	0.850–1.090
HGB	0.008	0.003	5.521	0.019	1.008	1.001–1.015
PLT	0.001	0.001	1.193	0.275	1.001	0.999–1.003
Sinus arrhythmia	−1.278	0.181	49.786	0.000	0.279	0.195–0.397
Ectopic cardiac rhythm	0.254	0.200	1.623	0.203	1.290	0.872–1.907
Conduction block	0.187	0.226	0.685	0.408	1.205	0.775–1.875
Pre‐excitation syndrome	2.586	1.891	1.871	0.171	13.277	0.326–540.212
Pacemaker heart rate	0.605	0.505	1.435	0.231	1.831	0.681–4.926
PRO	0.633	0.186	11.585	0.001	1.884	1.308–2.713
KET	0.181	0.253	0.514	0.473	1.199	0.730–1.969
RBCU	0.316	0.192	2.716	0.099	1.371	0.942–1.996
WBCU	−0.065	0.181	0.128	0.721	0.937	0.657–1.337
B‐AMY	−0.003	0.001	6.025	0.014	0.997	0.994–0.999
Urine pH	−0.442	0.122	13.234	0.000	0.643	0.506–0.815
RESP	−0.081	0.045	3.332	0.068	0.922	0.845–1.006
TEMP	−0.030	0.164	0.034	0.854	0.970	0.703–1.339
NIBPS	−0.008	0.005	2.832	0.092	0.992	0.983–1.001
SPO2	0.020	0.024	0.672	0.412	1.020	0.973–1.069
ALT	−0.001	0.002	0.490	0.484	0.999	0.996–1.002
GGT	0.003	0.001	28.046	0.000	1.003	1.002–1.005
ALP	−0.004	0.001	14.613	0.000	0.996	0.994–0.998
TP	−0.873	0.100	76.370	0.000	0.418	0.343–0.508
ALB	0.663	0.103	41.873	0.000	1.941	1.588–2.373
GLOB	0.850	0.101	71.413	0.000	2.340	1.921–2.850
TBIL	0.035	0.011	10.936	0.001	1.036	1.015–1.058
DBILI	−0.047	0.015	9.825	0.002	0.954	0.926–0.982
CR	−0.023	0.025	0.810	0.368	0.978	0.930–1.027
Urea	0.296	0.019	240.757	0.000	1.345	1.296–1.396
UA	−0.004	0.001	17.006	0.000	0.996	0.994–0.998
BNP	0.000	0.000	22.269	0.000	1.000	1.000–1.000

### 3.3 Correlation Analysis

Correlation analysis is the analysis of variables to measure the degree of correlation between two variable factors. The correlation among the features is evaluated by using Spearman's coefficient, and the significance of the correlation is tested. In heat maps, features with strong correlations are in dark red (positive correlation) or dark blue (negative correlation). If the *p* value of the significance test between two features is less than 0.001, it is marked as “***”. If 0.01 ≤ *p* < 0.05, it is marked as “*”. If *p* > 0.05, there is no marker, which means that the correlation is not significant.

A simpler model is always more stable and preferred. Inclusion of highly correlated variables may lead to numerical instability, masking interactions between different features, colluding the interpretability of an ML model and overfitting. Thus, it is always preferred to exclude one of the two correlated variables. If the Spearman's correlation coefficient *ρ* > 0.8 of the two features, the correlation between the two features is very strong and there is multicollinearity. Hence, one of the two features with *ρ* > 0.8 is usually eliminated in this paper. At the same time, in the process of significance test, for the features with no significant correlation, that is, *p* > 0.05, these features are eliminated in this paper.

**Figure 2:**
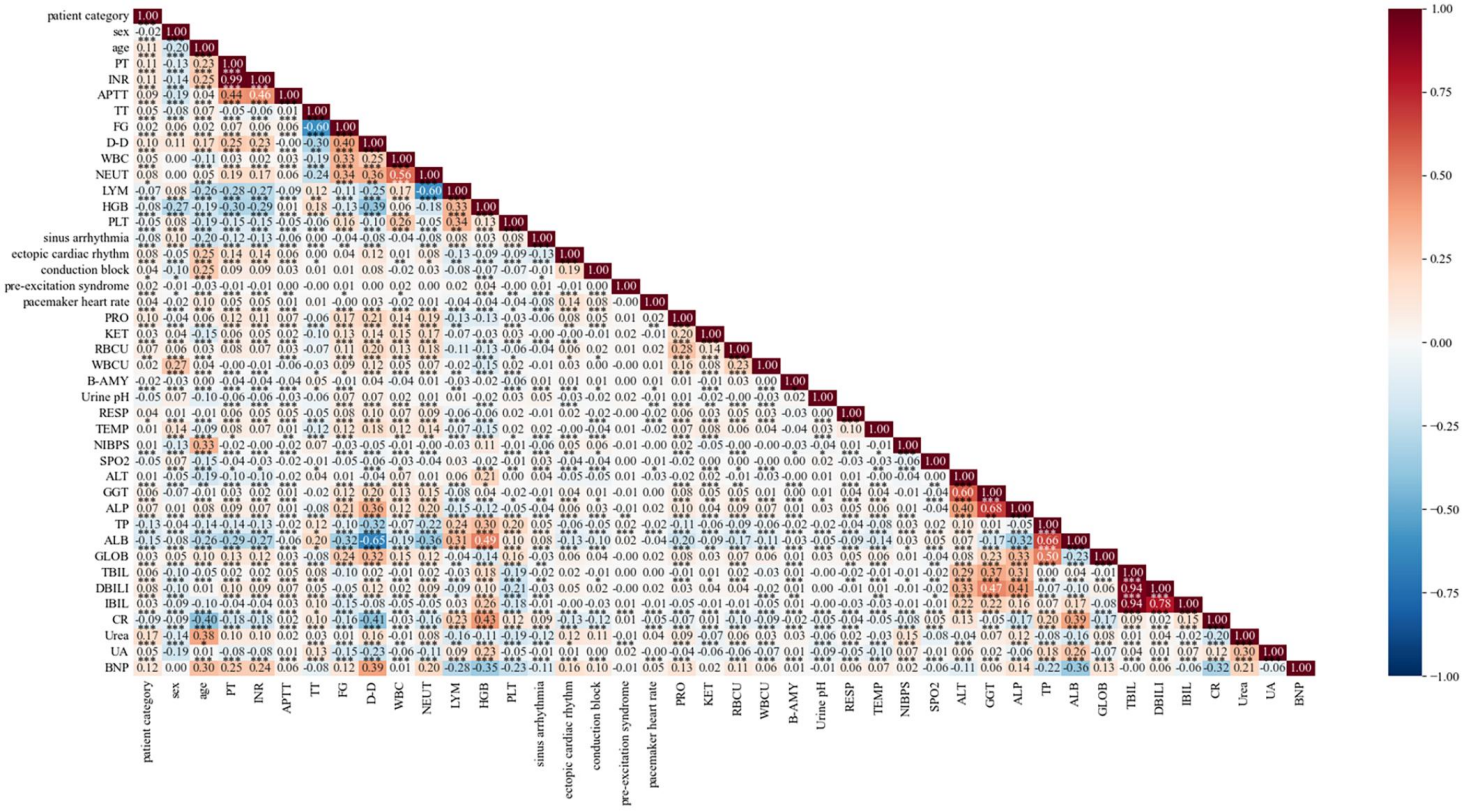
Correlation heat map.

From the heat map (Figure 2), we can see that the variables in different categories are weakly correlated, and only some of the variables in the same category show a strong correlation with each other.

#### 3.3.1 Liver Function

Overall, there is a strong correlation among the variables of liver function. DBIL and TBIL show a strong positive correlation with a significant relationship (*ρ* = 0.94, *p* < 0.001), while IBIL and TBIL also exhibit a strong positive correlation with a significant relationship (*ρ* = 0.94, *p* < 0.001). Additionally, considering the correlation between DBIL and IBIL (*ρ* = 0.78, *p* < 0.001) is close to 0.8, the decision is made to keep TBIL and remove DBIL and IBIL from the analysis. In the significance test with patient outcomes, since B‐AMY is not significant (*ρ* = −0.02, *p* > 0.05), this feature is removed.

#### 3.3.2 Urine Routine and Renal Function

Overall, there is a strong correlation among the variables of urine routine and renal function, so it is unlikely to encounter multicollinearity issues which may affect the model construction. At the same time, all variables show significant correlations with patient outcomes. Therefore, all these features are retained in this paper.

#### 3.3.3 Coagulation Function

The correlation between the variables of coagulation function is weak, and only a few variables have a certain intensity of correlation. Both PT and INR show strong positive correlations with significant relationships (*ρ* = 0.99, *p* < 0.001). As INR is a derived value calculated by experts to standardize PT and account for the sensitivity of reagent, it has more information. Therefore, only INR is kept, and PT is removed.

#### 3.3.4 Vital Signs

The correlation between the variables of vital signs is weak. In the significance test with patient outcomes, NIBPS (*ρ* = 0.02, *p* > 0.05) and RESP (*ρ* = 0.01, *p* > 0.05) are found to be nonsignificant, so these two variables are removed.

#### 3.3.5 Blood Routine Examination

The variables in the blood routine examination exhibit a certain level of strength in their correlations with each other, but the Spearman's correlation coefficients of them are not greater than 0.8. Therefore, all features are retained.

#### 3.3.6 ECG

The correlation between the variables of ECG is weak. In the significance test with patient outcomes, pre‐excitation syndrome (*ρ* = 0.01, *p* > 0.05) is found to be nonsignificant, so it is removed.

Finally, a total of 33 features are screened (25 continuous and 8 categorical).

## 4 ML Methodology

### 4.1 Supervised ML Approach

Predictive classifiers based on the training set are developed by using the following supervised ML methods: (1) AdaBoost, (2) XGBoost, (3) RF, (4) LR. Boosting is a classification algorithm that can promote weak learners to strong learners. AdaBoost and XGBoost are the representatives of boosting. RF represents traditional ML algorithms and is also the most frequently used algorithm in various prediction studies. It is an ensemble learning method based on Bagging. LR represents the traditional regression model associated with statistical analysis. It is a generalized linear regression analysis model.

The complete data set is randomly divided into a training set and a testing set with a ratio of 7:3. To address the adverse effects of data imbalance in the data set (where the ratio of minority sample to majority sample is 320:24 829), the synthetic minority over‐sampling technique (SMOTE) is adopted. SMOTE can help to balance the positive IHCA samples by generating synthetic examples to match the number of negative samples, thus further enhancing the reliability of the model. To eliminate data leakage and ensure unbiased model evaluation, SMOTE was strictly implemented only on the training subset within each fold of fivefold cross‐validation, and never applied to the entire data set before splitting. The independent test set remained completely untouched throughout the resampling process. This procedure ensures that model evaluation is performed on genuine unseen data, preventing artificial inflation of performance metrics.

For each model, the grid search approach is conducted to obtain the optimal hyperparameters. The best combination of hyperparameters is achieved through a fivefold cross‐validated grid search, with the objective of maximizing the AUROC.

### 4.2 Model Validation

The performance of each model is verified in the testing set. Model performance was estimated using AUROC, AUPRC, accuracy, recall, precision, and specificity. In order to interpret the final predictive model, the SHapley Additive exPlanation (SHAP) is adopted which is proposed by Lundberg and Lee in 2017 [20]. SHAP can explain any ML model's output by calculating the impact of each feature on model prediction based on game theory. SHAP helps us to understand which feature is the most important to model prediction, and the positive or negative direction of the feature impact (Table 4).

**Table 4:** Model performance in predicting inpatient mortality in patients with IHCA.

Algorithm	Feature selection method	AUROC (95% CI)	AUPRC	Accuracy	Recall	Precision	Specificity
AdaBoost	Raw data	0.975 (0.969–0.977)	0.589	0.982	0.762	0.428	0.986
	By Student's *t*‐test and Chi‐square test	0.971 (0.969–0.973)	0.568	0.982	0.771	0.422	0.985
	By regression analysis	0.968 (0.965–0.972)	0.535	0.975	0.781	0.329	0.978
	By correlation analysis	0.971 (0.966–0.974)	0.542	0.981	0.762	0.402	0.984
RF	Raw data	0.979 (0.978–0.981)	0.600	0.983	0.724	0.429	0.986
	By Student's *t*‐test and Chi‐square test	0.980 (0.977–0.979)	0.564	0.982	0.714	0.421	0.986
	By regression analysis	0.978 (0.976–0.980)	0.567	0.979	0.800	0.375	0.981
	By correlation analysis	0.979 (0.977–0.980)	0.575	0.982	0.695	0.420	0.986
LR	Raw data	0.961 (0.955–0.964)	0.395	0.950	0.829	0.193	0.951
	By Student's *t*‐test and Chi‐square test	0.954 (0.953–0.963)	0.403	0.947	0.857	0.190	0.948
	By regression analysis	0.964 (0.962–0.968)	0.410	0.951	0.857	0.203	0.952
	By correlation analysis	0.968 (0.966–0.969)	0.413	0.950	0.876	0.202	0.951
XGBoost	Raw data	0.987 (0.984–0.988)	0.763	0.992	0.695	0.723	0.996
	By Student's *t*‐test and Chi‐square test	0.980 (0.977–0.983)	0.736	0.992	0.657	0.711	0.996
	By regression analysis	0.984 (0.981–0.985)	0.723	0.991	0.714	0.670	0.995
	By correlation analysis	0.985 (0.981–0.986)	0.772	0.992	0.667	0.729	0.997

## 5 Results

### 5.1 Model Classification Analysis

Overall, across all four different ML approaches (AdaBoost, XGBoost, RF, and LR), the XGBoost model shows the best performance in this paper. RF and AdaBoost show about the same performance, and LR has an excellent recall performance, although it performs poorly in metrics such as AUROC and precision.

From the feature selection perspective, each feature selection method brings different effects in different classification models.

RF, Adaboost, and XGboost trained models using raw data with the best performance overall, while LR trained models using data filtered by correlation analysis better than the other three. At the same time, some feature selection methods have an impact on the improvement of a certain index of the model, such as the use of regression analysis to filter out the data of the model trained by the recall index have been significantly increased. In clinical settings, it is more important to identify patients who are likely to experience IHCA from a group of patients, so recall is an important metric for the model.

### 5.2 Feature Importance

**Figure 3:**
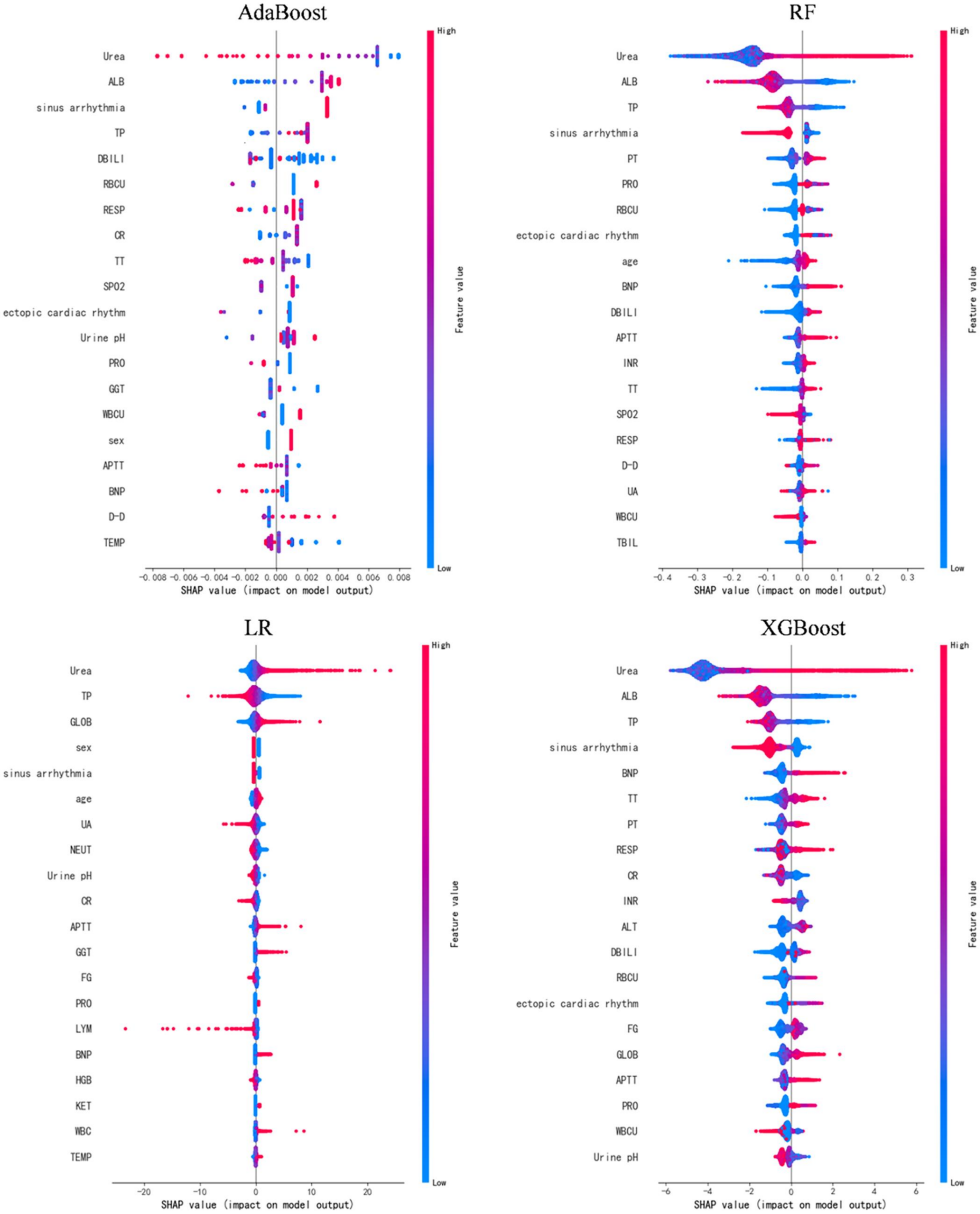
SHapley Additive exPlanation of the best classifiers of each ML model in the test set.

The best of the four classifiers trained by each ML model is selected for SHAP analysis (Figure 3).

The top 20 variables with the most influence on the model are shown on the *y*‐axis in rank order. By observing the top 10 feature rankings in each model, we can notice that urea, ALB, sinus arrhythmia, and TP are the most important variables, especially urea. However, urea has a missing rate of 80%, and it is prudent to look at the distribution of its data before and after filling, and we found that filling did not change the trend of its data distribution.

According to the regression analysis, correlation analysis, and SHAP analysis, a higher urea value (*B* = 0.296, *ρ* = 0.17) is associated with a higher risk of IHCA, while a lower ALB value (*B* = 0.663, *ρ* = −0.12) and a lower TP value (*B* = −0.873, *ρ* = −0.17) are associated with a higher risk of IHCA. Patients with sinus arrhythmia (*B* = −1.278, *ρ* = −0.08) on ECG have a lower probability of experiencing IHCA.

## 6 Discussions

IHCA is an adverse event associated with high mortality, and the incidence of IHCA is increasing year by year [21, 22]. The management of IHCA consumes a lot of medical resources [23]. Therefore, it is very important to develop an early warning model of IHCA that can support clinical decision‐making as soon as possible. Several risk models were published to address this clinical dilemma over the years. However, there is still little evidence supporting clinical decision‐making [24], and no model has been implemented in clinical practice up to now [25].

In this study, we try to develop a model to predict whether the IHCA occurs in patients based on the data of patients who have just been admitted to the hospital. Three feature selection methods (Student's *t*test and Chi‐square test, regression analysis and correlation analysis) and four ML models (AdaBoost, XGBoost, RF, and LR) are adopted in this paper. Each ML model is trained and evaluated using raw and feature‐selected data. As a result, 16 models are obtained. After horizontal and vertical comparison and model analysis, the main findings are as follows: (1) Compared with RF, AdaBoost, and LR, the XGBoost model demonstrates the best model performance. (2) Different feature selection methods have different effects on different ML models, so selecting the appropriate feature selection method is of great significance to further improve the performance of the model. (3) Feature selection using correlation analysis improves model recall significantly. (4) Among the 41 features included in the study, four features, including urea, ALB, sinus arrhythmia, and TP, are the most significant predictors. This prediction model is developed based on routine admission data that are universally collected in clinical practice, with no need for additional specialized examinations. It can be used as a supplementary decision‐support tool at the early stage of hospitalization: clinicians can perform preliminary risk stratification for IHCA based on the model's output, to timely identify patients at high risk, and make individualized adjustments to monitoring intensity and clinical management plans. This design ensures the model's clinical translation potential.

Different from most studies, the XGBoost model has the best performance, while the RF model had the best results in most studies. Nishq et al. compared 17 ML methods, including ANN and LSTM, and finally found that RF has the best performance (accuracy = 95.65%) [26]. In contrast to deep learning and neural networks, RF has received mixed reviews. In the study conducted by Jiang et al. [17], the performance of three ANN models (AUROC: 0.929, 0.933, 0.936) was superior to RF (AUROC: 0.923), while in the study by Hong et al. [27], the RF model (AUROC 0.97; AUPRC 0.86) was better than the ANN model (AUROC 0.95; AUPRC 0.82). There are also existing studies that prove that XGBoost is the optimal model. The study by Marta Fernandes et al. [28] proved that XGBoost exhibited good calibration properties and yielded areas under the receiver operating characteristic and precision‐recall curves of 0.96 (95% CI 0.95−0.97) and 0.31 (95% CI 0.26−0.36).

Many studies related to IHCA now favor the use of vital sign parameters only, as they are easier to monitor and measure and more relevant to medical practice. Some of the data in vital signs, such as respiration rate and blood pressure, have been shown to change before CA occurs [29], but models based only on vital sign parameters in existing studies have not been very predictive [8, 30, 31]. This suggests that for the prediction of IHCA, we may not be able to predict accurately using a single class of parameters, and we need multimodal data to do so, and we need to try to find some other easy‐to‐observe data for experimentation to build a model that is closer to the actual use and more accurate.

Unlike most studies, this study focuses on predicting IHCA occurrence based on data collected shortly after patient admission. In other studies, the prediction time window varies widely, ranging from 1 year before cardiac arrest to just a few seconds prior to the event. Additionally, many studies divide the prediction period into multiple intervals and report model performance for each separate time window [32]. This study focuses exclusively on the early admission window, which avoids the reliance on dynamic monitoring data after hospitalization and further ensures the simplicity and feasibility of the model in clinical practice. Indeed, one potential challenge remains that the model's prediction accuracy may be affected by unpredictable acute clinical deterioration of patients during hospitalization.

There are still limitations in this study. First, the missing rate of clinical features in this study remains non‐negligible. Although we balanced missing rate control and sample size retention during data screening, only 28 of the included features had a missing rate below 50%. Second, the predictive performance metrics of our model are more favorable than those reported in some previous similar studies, which may be partially attributed to the rigorous KNN imputation strategy we adopted for missing values. However, there is potential inherent bias in real‐world clinical data: patients with more severe conditions at admission usually undergo more comprehensive examinations and have more complete medical records, while patients with milder conditions tend to have more missing feature values. Although we performed KNN imputation stratified by IHCA outcome to minimize this bias, the potential impact of missing value imputation on model predictive performance cannot be completely ruled out. Third, only four ML algorithms and three feature selection methods were included in this study, which may lead to certain limitations and contingency in the results. Finally, according to the existing research, continuous observation data had a better effect on the prediction of IHCA [27, 33]. We only used the data at a certain time. If continuous observation data is used, the performance of the model may be improved. Moreover, we only evaluated whether patients developed IHCA as an outcome, and in future studies, we will evaluate other non‐clinical outcomes and long‐term outcomes.

The good news for us now is that with the development of medical technology, although the incidence of IHCA is increasing, the survival rate has also improved [21]. However, marked differences in the extent of survival improvement between different hospitals were observed [34]. This reminds us that the hospital process of care and the quality of the staff may be important [35]. For further studies, the increased comorbidity burdens of patients suffering cardiac arrests and the increased complexity of the disease may be a challenge. In order to further improve the treatment status of IHCA, we need to build an IHCA prediction system that can support clinical decision‐making and find a more standardized and reasonable hospital process of care [24].

## Consent

Patient consent was waived because the research involves no more than minimal risk and could not be practicably carried out without a waiver.

## Conflicts of Interest

The authors declare no conflicts of interest.

## Data Availability

The data presented in this study are available on request from the corresponding author.
